# Comparison of Critical Thinking among undergraduate medical students of Conventional and Integrated curricula in Twin Cities

**DOI:** 10.12669/pjms.38.6.5409

**Published:** 2022

**Authors:** Ume Sughra, Ambreen Usmani

**Affiliations:** 1 Dr. Ume Sughra, Associate Professor, Al-Shifa School of Public Health, Research Associate, Al-Shifa Research Centre, Assistant Editor, Al-Shifa Journal of Ophthalmology, Al-Shifa Trust Eye Hospital, Rawalpindi, Pakistan; 2 Prof. Dr. Ambreen Usmani, MCPS-HPE. Program Director, College of Physicians and Surgeons Pakistan, Karachi, Pakistan

**Keywords:** Critical thinking, Conventional, Integrated, Curriculum, Undergraduate, Medical, students

## Abstract

**Objectives:**

To compare critical thinking of undergraduate medical students of institutes following traditional and integrated curriculum at Twin cities.

**Methods:**

The current cross-sectional study was conducted in medical colleges of Twin Cities from February 2021 till August 2021. Two medical colleges one with conventional and other with integrated mode of curriculum were included. One hundred medical students were selected by simple random sampling from each conventional and integrated medical college. Free critical thinking test tool was used for data collection. The tool was composed of five sections, Arguments, Assumptions, Deductions, Inferences and interpreting information. Data entry and analysis was done by using SPSS version 20. Chi-Square test of independence was run to determine the association of critical thinking with type of curriculum. Independent sample t-test was applied to find out the mean difference in the critical thinking of medical students following the two different curriculums.

**Results:**

In current study 200 students were included. Majority were females (n= 155, 77.5%). The overall percentage of good critical thinking was found to be 49%. Majority of the students (n=57, 58.2%) having good critical thinking were found associated with integrated curriculum (p < 0.024, OR= 0.524, 95% CI= 0.3 - 0.92). There was statistically significant difference of critical thinking between institutes following two different curriculum strategies. Total critical thinking score was also found statistically significantly [MD= 5.00, 95% CI, (-1.05-8.96), p<0.013], more with integrated curriculum (133.48±15.6) as compared to conventional curriculum (128.47 ± 11.43).

**Conclusion:**

Critical thinking was found high among the students with the integrated curriculum as compared to the conventional.

## INTRODUCTION

Curriculum word is derived from the Latin word “curricula”, which means a race or the course of race. It is also described as a course for the students to gain their desired learning outcomes.^1^ Critical thinking, dating back to Socrates, is qualified as “ethical power guiding to virtue” and “logical way of thinking which guides our attitudes”. It is very effective for problem solving and decision making by analyzing complex data, evaluating situations and actions, and implementing the most appropriate actions.^2^

The ability to use logical reasoning to make decisions and solve problems while avoiding emotion driven evaluations and improvement of one’s own thought process. It helps in strengthening of cognitive actions and develops creative solutions to the problems.^3^

In revised cognitive process Bloom Taxonomy, the knowledge is renamed as Remember and Comprehension with understanding. Application, Analysis and Evaluation were retained but changed into verb form as apply, analyze and evaluate.^4^,^5^

Weidman B et al. did a theoretical review of curriculum integration in medical education. They evaluated and referred, knowledge boom, fragmented teaching methods, concerns over curriculum relevancy and lack of relationships and connections among disciplines as potential reasons for a shift towards integrated curriculum.^6^

Curriculum integration is an approach that brings together knowledge, attitude, values and skills from within the subject or across the subject areas to develop a strong understanding of the key concepts. It is essential to have skills to think clearly and rationally about what to do or what to believe for medical practices. Critical thinking is a proven factor that plays an influential role in routine medical practices such as choosing treatment plans, making accurate diagnosis and reducing medical errors.^7^-^9^

Currently both traditional and integrated curriculums are being followed and practiced in medical schools of Pakistan. So, there is a great need to do research and add on evidence to fill the existing gaps present between the type of curriculum chosen and required skill development among healthcare professionals.

**Fig.1:**
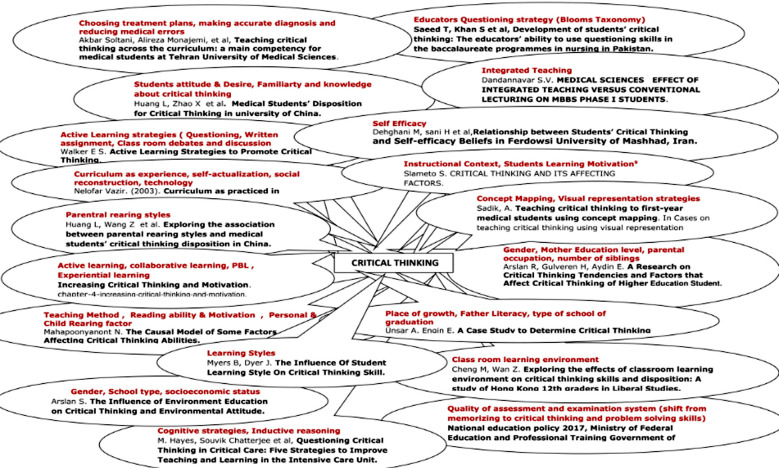
Factors effecting Critical Thinking identified from Literature review.

It is therefore suggested that there is a great need to study and analyze the current curriculums and teaching practices in our medical schools to emphasize the importance of developing the clinical reasoning skills among our health care professionals. This will help in improving our healthcare system and better clinical practices in the society. In current study factors associated with critical thinking have been identified from literature review and depicted in Fig.1.

This research would help to differentiate between conventional and integrated curriculum in stimulating critical thinking among undergraduate medical students. In the light of evidence reforms might be taken by the educationist and policy makers for the existing curriculum. Furthermore, a successful curriculum will also reduce the overlap of different disciplines and subsequently reduce overall teaching hours, giving students greater time to spend on autonomous learning and to practice independent thinking. The objective of current study was to compare critical thinking among undergraduate medical students of Conventional and Integrated curricula in Rawalpindi and Islamabad cities.

## METHODS

The cross-sectional study was conducted on 200 undergraduate medical students studying in medical colleges of twin cities Rawalpindi-Islamabad from February 2021 till August 2021.

The sampling strategy was simple random sampling. Sample size was calculated by using formula z^2^pq/e^2^, with expected critical thinking Prevalence (P) of 64%,^10^ q is 100-p=36%, Precision level was kept at 7. After putting all the values in the formula z^2^pq/e2 = 1.96*1.96*64*36/7*7, sample size came out to be 188. The final sample size was taken as 200 to adjust the non-response. Undergraduate medical students of both genders were included. There is total 12 medical colleges in twin cities out of which three are in public sector and nine are in private sector. Medical colleges with conventional and integrated teaching curriculums were separated after visiting and checking their curriculum. One medical college with conventional and one with integrated curriculum were selected after examining the curriculums because most institutes were following hybrid curriculums. The institute following integrated curriculum was at the eight level of Harden’s Integration Ladder that was “Complementary approach”.^11^ After selection of medical colleges one hundred students from conventional and one hundred from integrated medical colleges in their clinical years were included using computer generated random numbers.

Prior to data collection, appropriate permissions were obtained from the ethical review committee of the institute (Pakistan Institute of Ophthalmology, Al-Shifa Trust Eye Hospital Rawalpindi) (ERC No: 67/AST-20). Verbal informed consent was taken by each student.

Self-administered questionnaire was used for Data collection. Pilot study was done on 10% of sample size (n=20). Free critical thinking test tool was used for obtaining data regarding critical thinking (https://www.assessmentday.co.uk/CriticalThinkingTest-Questions.pdf). It was a validated and reliable tool with a reliability score of 0.778 Cronbach’s Alpha value. (Table-I) This tool contains 80 questions. The tool was composed of five sections. First section was “Arguments” which contained eight statements with 04 questions each. Each question of every statement was based on weak and strong agreement. True answer was coded as one and wrong answer was coded as 0. Second section was Assumptions. This section contained four statements each followed by proposed assumptions. The responses of each assumption were as “Assumptions Made” and “Assumptions not made”. Correct answer was given code-2 and incorrect was given code- 1. Third section was Deductions. This section contained seven statements and each statement was followed by suggested conclusions. The responses of each conclusion were as “conclusion follows” and “conclusion does not follow”. Correct answer was given code-2 and incorrect was given code-1. Fourth section was Inferences. This section contained three statements. From each statement further inferences were drawn. The responses of each inference were as “True, probably true, more information required, probably false and false”. The correct answer was given a code of 1 and wrong answer was given a code of 0. Fifth Section was interpreting information. It contained four statements. Each statement was followed by conclusions. The correct conclusion was given a code of 1 and incorrect conclusion was given a code of 0. All sections were computed individually to calculate mean total critical thinking score. Data entry and analysis was done by using SPSS version 20. Descriptive analysis was done to determine frequencies and percentages for qualitative variables and means and standard deviations were reported for quantitative variables. After checking normality of outcome variable, mean, median and 5% trimmed mean of both groups were found to be same with a bell-shaped histograms of both groups. Independent sample t-test was applied to find out the mean difference in the critical thinking of medical students following the two different curriculums.

**Table I:** Reliability analysis of the tool.

*Scale Statistics*			

*Mean*	*Variance*	*Std. Deviation*	*N of items*

131.2074	212.155	14.56553	80

*Reliability Statistics*			

*Cronbach’s Alpha*		*N of Items*	

0.778		80	

## RESULTS

**Fig.2:**
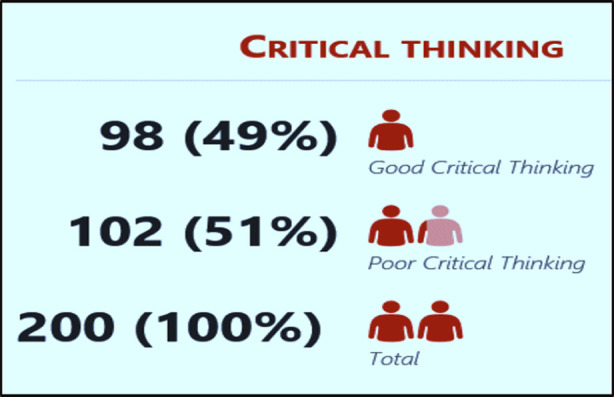
Overall percentage of Good Critical thinking.

In current study 200 students were included. Majority were females (n=155, 77.5%). Most of the students were living in hostels (n=130, 65%). Mean age of the students was 22.8±0.36 (Table-II). The overall percentage of good critical thinking was found 49% (n=98) Fig 2.

**Table II:** Socio demographic variables.

Gender	Frequency (n=200)	Percent (100)
Male	45	22.5%
Female	155	77.5%

*Mode of teaching*	*Frequency (n=200)*	*Percent (100)*

Integrated	100	50%
Conventional	100	50%

*Living status*	*Frequency (n=200)*	*Percent (100)*

Hostelite	130	65.0%
Day Scholar	70	35.0%

**Fig.3:**
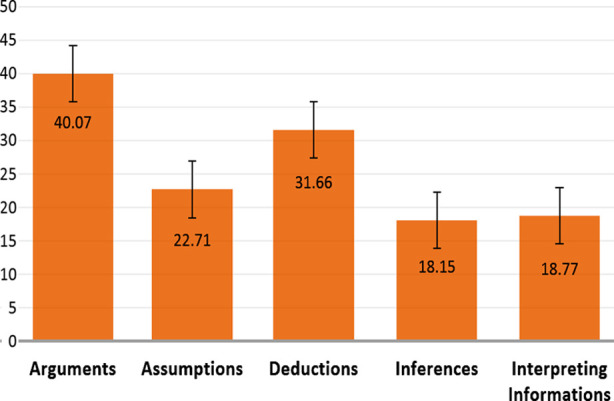
Mean and confidence intervals of Subsections of Critical Thinking Scale.

Chi square test of independence showed the association between critical thinking and mode of curriculum. Majority of the students (57, 58.2%), out of ninety-eight having good critical thinking were found associated with integrated curriculum (p-value= 0.024, OR= 0.524, 95% Confidence Intervals= 0.3 - 0.92). The overall total mean score of critical thinking was found to be 131.38±1.0. The mean scores of sub sections of critical thinking were presented in Fig.3.

There was statistically significant difference of critical thinking between mode of curriculum (MD= 5.00, 95% CI, (-1.05-8.96), p= 0.013]. Total critical thinking score was found more in integrated mode of learning (133.48±15.6) as compared to conventional (128. 47 ± 11.43) (Table-III). There was also statistically significant difference (p<0.05) of components of critical thinking tool including, arguments (MD=-0.84), assumptions (-0.61), deductions (-0.38), inferences (-5.95) and interpreting information (-0.12) between modes of curriculum (Table-IV).

**Table III:** Association between Critical thinking and Mode of Curriculum.

	Total critical thinking (n=200)		Total	p-value	Odd Ratio	95% CI

	Poor	Good				
Integrated	43 (42.2)	57(58.2)	100	0.024	0.524	0.3 - 0.92
Conventional	59(57.8)	41(41.8)	100			

Total	102 (100)	98(100)	200			

**Table IV:** Comparison of critical thinking between integrated and conventional curriculum (n=200).

Critical Thinking	Overall Mean±SD	Integrated Mean±SD	Conventional Mean±SD	Mean Difference	p-value	95% Confidence Interval	

						Lower	Higher
Arguments	40.07±0.11	39.71±1.68	40.55±1.40	-0.84	.000	-1.28	-0.39
Assumptions	22.71±0.10	22.45±1.75	23.07±1.10	-0.61	.005	-1.04	-.18
Deductions	31.66±0.10	31.82±1.52	31.44±1.54	0.38	.080	-0.04	.82
Inferences	18.15±0.99	20.65±15.98	14.70±9.96	5.95	.003	2.05	9.84
Interpreting information	18.77±0.23	18.82±3.16	18.70±3.45	0.12	.791	-0.80	1.05

Total Critical thinking	131.38±1.0	133.48±15.6	128.47±11.43	5.00	0.013	1.05	8.96

**Fig.4:**
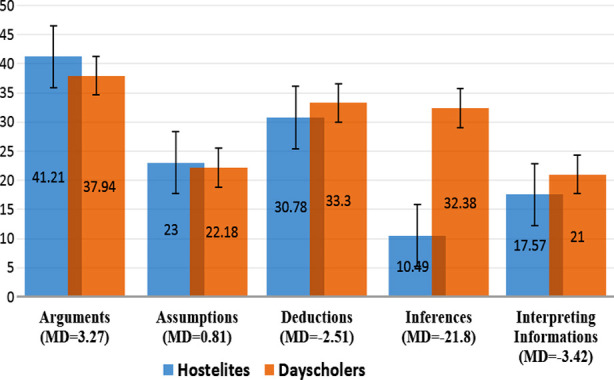
Graphical Presentation of Unpaired t test to compare subsections of critical thinking between hostelites and day scholars (α ≤0.001).

There was statistically significant difference of critical thinking with living status (Hostelites/ Day scholars) [(MD= -23.74, 95% CI (-26.24-21.24)]. Total critical thinking score was also found more among day scholars (146.81±8.75) as compared to hostelites (123.06±8.42) (Figure: 03) with a statistically significant difference (p < 0.05) of components of critical thinking tool subscales including, arguments (MD= 3.27), assumptions (0.81), deductions (-2.51), inferences (-21.8) and interpreting information (-3.42) (Fig: 4).

There was no statistically significant difference of total critical thinking between gender (p= 0.1). The components of critical thinking were also not found statistically significant with both genders individually (P-value>0.05) (Table-V).

**Table V:** Comparison of Critical thinking among Gender (n=200).

Critical Thinking	Overall Mean±SD	Male Mean±SD	Female Mean±SD	Mean Difference	p-value	95% Confidence Interval	

						Lower	Higher
Arguments	40.07±0.11	40.37±1.49	39.98±1.65	0.39	0.14	-0.14	0.93
Assumptions	22.71±0.10	22.86±1.32	22.67±1.60	0.19	0.45	-0.32	0.71
Deductions	31.66±0.10	31.48±1.50	31.71±1.55	-0.22	0.386	-0.74	0.28
Inferences	18.15±0.99	15.57±10.91	18.90±14.79	-3.32	0.163	-8.01	1.35
Interpreting information	18.77±0.23	18.06±3.42	18.98±3.22	-.91	0.100	-2.0	0.17

Total Critical thinking	131.38±1.0	128.37±12.32	132.25±14.61	-3.87	0.10	-8.59	0.84

## DISCUSSION

In current study critical thinking was found high among the students with the integrated mode of curriculum as compared to the conventional curriculum. The finding of current study was in accordance with studies conducted by Ezequiel Od et al. and Yadav P et al. who reported better critical thinking abilities of students with integrated mode of teaching as compared to conventional teaching strategies.^12^,^13^ Similar studies conducted by Pu D et al. and Ibrahim et al. also found that traditional teaching does not significantly improve critical thinking and problem solving skills but integrated learning strategy enhanced the problem solving skill and critical thinking scores significantly.^2^

In a study conducted in China by Huang L et al. reported that after one year of conventional teaching method overall percentage of medical students with better critical thinking scores increased from 66.96% to 73.21% to 66.96% .^14^ Masic I et al. identified the factors influencing the critical thinking abilities of students. The greatest influence on critical thinking was found to be the teaching methods along with availability of resources for the implementation of integrated curriculum .^15^,^16^ All these study results are found to be similar with the present study results.

In present study there was no statistically significant difference in critical thinking of males and females, although female CT scores were higher as compared to males. Findings of a study conducted by Ivanna S et al. are in accordance with the current where no statistically significant difference of CT was found between gender.^17^-^19^ Allahi E et al. also reported gender as one of the significant predictors of critical thinking. They also reported other factors such as faculty, department, year, parents’ education level, parents’ occupation, residential area, and number of siblings with high disposition of critical thinking (R=0.279, R2 =0.078, p<.00). Similar studies found higher critical thinking scores in females as compared to males. This might be due to the social structure and social learning that may help girls grow up as individuals who have to deal with more problems as compared to men. They become more attentive to details, have more confidence in themselves and their thinking processes and possess the abilities to think flexibly as well.^20^

Current study found that students who are hostelites had low critical thinking as compared to the day scholars. Study conducted by Mattick & Knight revealed that students who spend more time for learning independently are more likely to have better problem solving.^21^ Shaheen N et al. also reported critical thinking problems of students living in hostels and away from their families.^22^ Rehman K et al. reported no difference of academic performance in medical professional exam but day scholars were found to be better than their other counterparts.^23^,^24^

The country’s education system needs to identify all the possible barriers while inculcating a change in the thinking style, research, deepening critical thinking, and also a change in teachers’ attitudes toward curriculum designing (goals, objectives, learning content, instructional designs and evaluation methods); more so, it is suggested that proper attention should be paid to the need to develop and utilize critical thinking skills in the learners’ education.^25^

The current study results suggest that medical curriculum should be taught in an integrated manner to stimulate critical thinking skills among future healthcare providers. Inculcating critical thinking and problem-solving skills should be a major objective of medical teaching and curriculum.

### Limitations of the study

The most important is that it was carried out on a sample from two of the medical institutes so generalizability of the results should be done cautiously. The design of the study was cross-sectional so any change in critical thinking level could not be assessed from their entry point to medical institute until graduation. It is suggested that more comprehensive researches should be carried out with representation from all over the country to identify the associations of curriculum modes critical thinking levels among medical students.

## CONCLUSION

Critical thinking was found high among the students with the integrated mode of curriculum as compared to the conventional curriculum.
